# Health-related quality of life and recovery capital among recovery residents taking medication for opioid use disorder in Texas

**DOI:** 10.3389/fpubh.2023.1284192

**Published:** 2023-11-20

**Authors:** Elizabeth O. Obekpa, Sheryl A. McCurdy, Vanessa Schick, Christine M. Markham, Kathryn R. Gallardo, Johnny Michael Wilkerson

**Affiliations:** ^1^Department of Health Promotion and Behavioral Science, The University of Texas Health Science Center Houston, School of Public Health, Houston, TX, United States; ^2^Department of Management, Policy, and Community Health, The University of Texas Health Science Center Houston, School of Public Health, Houston, TX, United States; ^3^Department of Health Promotion and Behavioral Science, The University of Texas Health Science Center Houston, School of Public Health, Houston, TX, United States

**Keywords:** health-related quality of life, EQ-5D-5L, recovery capital, opioid use disorder, medication for opioid use disorder, recovery homes, sober living homes

## Abstract

**Background:**

Recovery from opioid use disorder (OUD) includes improvements in health-related quality of life (HRQOL) and is supported by recovery capital (RC). Little is known about RC and HRQOL among recovery residents taking medication for OUD. We described HRQOL and RC and identified predictors of HRQOL.

**Methods:**

Project HOMES is an ongoing longitudinal study implemented in 14 recovery homes in Texas. This is a cross-sectional analysis of data from 358 participants’ on HRQOL (five EQ-5D-5L dimensions—mobility, self-care, usual activities, pain/discomfort, and anxiety/depression) and RC (Assessment of Recovery Capital scores) collected from April 2021 to June 2023. Statistical analyses were conducted using T-, Chi-squared, and Fisher’s exact tests.

**Results:**

Most participants were 35 years/older (50.7%), male (58.9%), non-Hispanic White (68.4%), heterosexual (82.8%), and reported HRQOL problems, mainly anxiety/depression (78.4%) and pain/discomfort (55.7%). Participants who were 35 years/older [mean (SD) = 42.6 (7.3)] were more likely to report mobility and pain/discomfort problems than younger participants. Female participants were more likely to report pain/discomfort problems than male participants. Sexual minorities were more likely to report anxiety/depression problems than heterosexual participants. Married participants and those in committed relationships were more likely to report problems conducting self-care than single/never-married participants. Comorbid conditions were associated with mobility, pain/discomfort, and usual activities problems. Most participants reported high social (65.4%), personal (69.0%), and total (65.6%) RC. Low personal RC was associated with mobility (aOR = 0.43, CI = 0.24–0.76), self-care (aOR = 0.13, CI = 0.04–0.41), usual activities (aOR = 0.25, CI = 0.11–0.57), pain/discomfort (aOR = 0.37, CI = 0.20–0.68), and anxiety/depression (aOR = 0.33, CI = 0.15–0.73) problems. Low total RC was associated with problems conducting self-care (aOR = 0.20, CI = 0.07–0.60), usual activities (aOR = 0.43, CI = 0.22–0.83), pain/discomfort problems (aOR = 0.55, CI = 0.34–0.90), and anxiety/depression (aOR = 0.20, CI = 0.10–0.41) problems. Social RC was not associated with HRQOL.

**Conclusion:**

Personal and total RC and comorbid conditions predict HRQOL. Although the opioid crisis and the increasing prevalence of comorbidities have been described as epidemics, they are currently being addressed as separate public health issues. Our findings underscore the importance of ensuring residents are provided with interprofessional care to reduce the burden of comorbidities, which can negatively impact their OUD recovery. Their RC should be routinely assessed and enhanced to support their recovery and improve HRQOL.

## 1 Introduction

Substance use disorders (SUDs), including opioid use disorder (OUD), negatively impact the quality of life (QOL), physical, mental, and emotional states, and social interactions of individuals who use substances (1). The opioid epidemic in the United States (US) is worsening, with over 9 million individuals misusing opioids and 5.6 million individuals having an OUD in 2021 (2). Over 91,000 drug overdose deaths were reported in 2020, an increase of 31% from 2019, and nearly 75% of all overdose deaths were attributed to opioids (3). Opioid and stimulant use disorders and drug overdoses in the US were responsible for 15.03 million disability-adjusted life years between March 2020 and February 2022 (4).

There is an increasing interest in QOL for decision-making, especially for economic evaluations, and as an essential outcome in clinical care and SUD recovery (1, 5, 6), particularly as perceived health-related quality of life (HRQOL) is a stronger predictor of mortality and morbidity than objective assessments of health (7, 8). Individuals with current or past OUD have significant reductions in HRQOL (9). Individuals with SUDs seeking treatment have persistently lower HRQOL than those without SUDs (1). Positive changes in HRQOL among individuals with OUD are associated with recovery outcomes, including improvements in stable housing and decreases in illicit drug use (10).

Risk factors for OUD and opioid overdose deaths involving prescription and non-medical opioid use include mental and physical comorbidities and a history of SUD (11, 12). The prevalence of comorbid mental disorders is higher in adults with OUD compared to the general population (12, 13), with depression and anxiety being the most prevalent and associated with poorer HRQOL (14–17). Similarly, asthma is the most prevalent chronic disease related to opioid-related hospitalization, followed by obesity, liver disease, arthritis, cancer, and stroke (18). Patients with these chronic diseases often experience chronic pain (18) and significant reductions in their HRQOL (19–23). About 20.4% of U.S adults were living with chronic pain in 2016 (24), with researchers finding that individuals who reported chronic pain have problems conducting daily activities, mobility restrictions, worse health status, disability, and increased mortality risk (25–28).

Individuals who engage in the non-medical use of opioids frequently engage in polysubstance use, complicating the diagnosis and treatment of SUDs and accounting for most opioid-related overdose deaths (29). Most individuals engaging in polysubstance use have lower HRQOL (30) and develop comorbid SUDs (12). For instance, among individuals with an opioid (heroin) use disorder, about a quarter have an alcohol use disorder, more than 20% have a cocaine use disorder, and 12.3% have a marijuana use disorder (12). Other predictors of HRQOL among individuals with SUDs include sociodemographic characteristics, e.g., younger age, male gender, Caucasian race, marital status, employment, and higher educational qualifications, which positively correlate with higher HRQOL dimensions (1, 31–35).

Recovery from OUD is a multidimensional concept that includes improvements in mental and physical health and overall functioning following abstinence (36). Many individuals with OUD have poorer HRQOL than non-users (9, 37). Most literature on HRQOL emphasizes the impact of chronic diseases on an individual’s health and well-being rather than identifying predictors of HRQoL and resources that could support recovery from OUD and improve HRQoL (37, 38). Such research could help health planners, policymakers, and recovery resident operators plan and implement more effective recovery programs for OUD. This is critical as recovery from OUD is supported by medication for OUD (MOUD) and an individual’s ability to use resources for abstinence initiation and maintenance (39). However, despite the effectiveness of MOUD in treating OUD, decreasing illicit substance use, and improving retention in care, uptake remains low (40).

Recovery capital (RC) describes the entirety of resources individuals can use to support their recovery initiation and maintenance (39). RC comprises five resources: social, personal, physical, community, and cultural (41). However, social and personal RC may be stronger predictors of long-term recovery, with high social RC regulating the impact of low personal RC (42). Social RC is the benefits obtained from social networks and relationships that support recovery, including social support and social expectancies (41, 42). Personal RC is defined as individual characteristics, including self-efficacy, education/vocational skills, and mental and physical health (41, 42). Social and personal RC can be continually amassed or depleted over time as an individual’s opioid use or recovery impacts their personal and social functioning (43). Understanding RC in research and practice is critical, particularly as the more RC an individual possesses, the higher their perceived QOL may be.

The World Health Organization QOL (WHOQOL) dimensions are associated with social and personal RC among emerging adults in substance use treatment (43) and total RC in treatment and recovery samples (6). RC improves QOL by 22% among individuals with an SUD (44). High social RC is associated with greater QOL and enhanced health outcomes (43, 45). Personal RC, such as abstinence self-efficacy, is associated with greater QOL (46). Although research on RC and HRQOL among individuals with SUDs is growing, more attention needs to be paid to RC and HRQOL among individuals with OUD living in recovery homes. This study aimed to describe RC and HRQOL across five health dimensions (mobility, self-care, usual activities, pain/discomfort, and anxiety/depression) and identify predictors of the health dimensions among a sample of recovery residents taking MOUD. Specifically, we aimed to answer two research questions: (1) do participants differ in their RC and HRQOL levels when entering the recovery homes for OUD recovery support? (2) what are the predictors of HRQOL?

## 2 Methods

### 2.1 Design

Project HOMES (Housing for Opioid Medication-Assisted Recovery Expanded Services) is an ongoing longitudinal study of 14 level II (monitored homes, staffed with a paid house manager) and III (supervised homes, staffed with a paid house manager, Director/Administrator, and certified peer support) recovery homes (47) for persons in recovery for OUD and residing in five Texas cities. Eligibility includes a primary diagnosis of OUD, taking MOUD or willing to take before move-in, 18+ years, English or Spanish speaking, able and willing to consent, and agree to sign the Health Insurance Portability and Accountability Act (HIPAA)-compliant releases. Trained data collectors obtained written informed consent. Data analyzed were collected from April 2021 through June 2023. Ethical approval was granted by the institutional review board of the authors’ home institution. Participants received a $25 gift card for their time.

### 2.2 Measures

#### 2.2.1 Sociodemographic characteristics

Participants were asked about their age, race/ethnicity, sex at birth, sexual orientation, education, employment, and marital status (48). To ensure adequate statistical power in the regression models, dichotomous variables for race/ethnicity (non-Hispanic White and racial/ethnic minorities) and employment (employed and unemployed) were created.

#### 2.2.2 HRQOL dimensions

Health-related Quality of Life (HRQoL) was measured using the EQ-5D-5L instrument, which consists of a descriptive system that assesses participants’ self-rated health state in five dimensions - mobility, self-care, usual activities, pain/discomfort, and anxiety/depression (49). Participants indicate their functioning level in a given dimension. Each dimension has five response levels of severity - no problem (1), slight problem (2), moderate problem (3), severe problem (4), and extreme problem (5) (see Table 1). The EQ-5D-5L responses were dichotomized as no problems and any problems (slight, moderate, severe, and extreme) to change the health states into frequencies of reported problems (49). Responses were also combined to generate health state profiles ranging from full health (11111) to worst health (55555). Each value for a health state was linked to a value set, i.e., index values (weights) for the US. The HRQOL index values range from −0.281 to 1, with negative values representing health states worse than death, 0 representing a health state equivalent to death, and 1 representing full health (49). Sample *α* = 1.000.

**Table 1:** Self-reported 5Q-5D-5L problems by recovery capital.

5Q-5D-5L dimensions	Total	Total recovery capital			Social recovery capital			Personal recovery capital		
		Low 122 (%)	High 233 (%)	*p*-value	Low 123 (%)	High 232 (%)	*P*-value	Low 110 (%)	High 245 (%)	*P*-value
	n (%)									
Mobility				**0.021**			0.289			**<0.001**
No problems	291 (81.3)	91 (73.4)	200 (85.5)		96 (78.0)	195 (84.0)		78 (70.9)	213 (86.9)	
Slight problems	40 (11.2)	16 (12.9)	24 (10.3)		15 (12.2)	25 (10.8)		16 (14.6)	24 (9.8)	
Moderate problems	14 (3.9)	9 (7.3)	5 (2.1)		8 (6.5)	6 (2.6)		10 (9.1)	4 (1.6)	
Severe problems	10 (2.8)	6 (4.8)	4 (1.7)		4 (3.3)	6 (2.6)		6 (5.4)	4 (1.6)	
Unable to walk	–	–	–		–	–		–	–	
Self-care				**0.016**			0.387			**<0.001**
No problems	336 (93.8)	109 (87.9)	227 (97.0)		114 (92.7)	222 (95.7)		96 (87.3)	240 (98.0)	
Slight problems	10 (2.8)	7 (5.7)	3 (1.3)		5 (4.1)	5 (2.2)		7 (6.4)	3 (1.2)	
Moderate problems	8 (2.3)	5 (4.0)	3 (1.3)		3 (2.4)	5 (2.2)		6 (5.4)	2 (0.8)	
Severe problems	1 (0.8)	1 (0.8)	0 (0.0)		1 (0.8)	0 (0.0)		1 (0.9)	0 (0.)	
Unable to wash or dress	–	–	–		–	–		–	–	
Usual activities				**<0.001**			0.159			**<0.001**
No problems	307 (85.8)	95 (76.6)	212 (90.6)		100 (81.3)	207 (89.2)		82 (74.5)	225 (91.8)	
Slight problems	29 (8.1)	11 (8.9)	18 (7.7)		12 (9.8)	17 (7.3)		13 (11.8)	16 (6.5)	
Moderate problems	14 (3.9)	11 (8.9)	3 (1.3)		8 (6.5)	6 (2.6)		10 (9.1)	4 (1.6)	
Severe problems	4 (1.1)	4 (3.2)	0 (0.0)		2 (1.6)	2 (0.9)		4 (3.6)	0 (0.0)	
Unable to do	1 (0.3)	1 (0.8)	0 (0.0)		1 (0.8)	0 (0.0)		1 (0.9)	0 (0.0)	
Pain/Discomfort				**<0.001**			0.223			**<0.001**
No pain	166 (46.4)	43 (33.9)	124 (53.0)		50 (40.7)	116 (50.0)		34 (30.9)	132 (53.9)	
Slight pain	72 (20.1)	22 (17.7)	50 (21.4)		24 (19.5)	48 (20.7)		19 (17.3)	53 (21.6)	
Moderate pain	78 (21.8)	33 (26.6)	45 (19.2)		30 (24.4)	48 (20.7)		33 (30.0)	45 (18.4)	
Severe pain	26 (7.3)	16 (12.9)	10 (4.3)		12 (9.8)	14 (6.0)		14 (12.7)	12 (4.9)	
Extreme pain	13 (3.6)	9 (7.3)	4 (1.7)		7 (5.7)	6 (2.6)		10 (9.1)	3 (1.2)	
Anxiety/Depression				**<0.001**			**<0.001**			**<0.001**
None	86 (24.0)	10 (8.1)	76 (32.5)		15 (12.2)	71 (30.6)		10 (9.1)	76 (31.0)	
Slightly	105 (29.3)	29 (23.4)	76 (32.5)		33 (26.8)	72 (31.0)		25 (22.7)	80 (32.6)	
Moderately	104 (29.1)	42 (33.9)	62 (26.5)		43 (35.00)	61 (26.3)		35 (31.8)	69 (28.2)	
Severely	35 (9.8)	25 (20.2)	10 (4.3)		21 (17.1)	14 (6.0)		23 (20.0)	12 (4.9)	
Extremely	25 (7.0)	16 (12.9)	9 (3.8)		11 (8.9)	14 (6.0)		17 (15.4)	8 (3.3)	

Differences in counts result from missing data. Bolded values indicate statistical significance at *p*-value < 0.05.

#### 2.2.3 Polydrug use

Participants were asked about their illicit drug use or misuse in the past 90 days (48). The most frequently reported drugs used were street opioids (55.0%), followed by amphetamines (45.3%), methamphetamines (42.2%), benzodiazepines (38.8%), marijuana (37.5%), prescription opioids (26.0%), and cocaine (24.3%) (Supplementary Table S1). Participants were categorized as engaging in polydrug use based on the number of reported drugs used in the past 90 days.

#### 2.2.4 Hazardous drinking

Hazardous drinking was assessed using the Alcohol Use Disorders Identification Test (AUDIT) (50). The AUDIT measures the frequency of drinking, typical quantity, frequency of heavy drinking, impaired control over drinking, increased salience over drinking, morning drinking, guilt after drinking, blackouts, alcohol-related injuries, and others’ concerns about drinking in the past year. Participants with scores of 8 or above were categorized as having a high risk for hazardous drinking (sample *α* = 0.879).

#### 2.2.5 Comorbid diagnoses

Participants were asked about 36 comorbid diseases, including depression, anxiety, cancer, arthritis, stroke, and diabetes. Participants were categorized as having comorbid diagnoses or not based on the number of reported comorbid diagnoses.

#### 2.2.6 Recovery capital

The Assessment of Recovery Capital (ARC) is a 50-item, dichotomous (0 = disagree, 1 = agree) scale used to assess participants’ RC across two domains (6). Each domain has five dimensions. The social RC domain includes substance use and sobriety, citizenship and community involvement, social support, meaningful activities, and housing and safety. The personal RC domain has global psychological and physical health, risk-taking, coping and life functioning, and recovery experience. Participants were asked to indicate their agreement or disagreement with each statement. The sum scores were calculated to create scores for social and personal ARC ranging from 0 to 25 and a total ARC score ranging from 0 to 50. Using cutoff points described in Obekpa et al. (51), ARC scores were dichotomized as low or high social, personal, and total RC (sample α: total = 0.852, social = 0.713, and personal = 0.768).

### 2.3 Statistical analysis

Study variables were summarized using descriptive statistics (means, standard deviation (SD), frequencies, and percentages). EQ-5D-5L dimensions were compared by participant’s characteristics and ARC scores using T-, Chi-square, or Fisher’s exact tests. Variables significant at *p* < 0.1 were entered into multivariable logistic regression models to explore their relationships with each EQ-5D-5L dimension. Results of *p* < 0.05 were considered significant. Analyses were computed using Stata/MP 16 (52).

## 3 Results

The mean age in our sample was 36.0 (*SD* = 8.9). Most participants were 35 years or older (50.7%; mean age = 42.6, *SD* = 7.3), non-Hispanic White (68.4%), heterosexual (82.8%), male (58.9%), single or never married (62.1%), unemployed (66.0%; 5.4% were unemployed, disabled), attended some college or had a college degree (50.1%), and engaged in polydrug use in the past 30 days (75.5%). Only 8.7% had a high risk for hazardous drinking. The mean scores for social, personal, and total RC were 21.36 (*SD* = 3.50), 21.39 (*SD* = 3.60), and 42.75 (*SD* = 6.63), respectively (Table 2). The mean score for the mobility dimension was 1.28 (*SD* = 0.67), self-care 1.08 (*SD* = 0.37), usual activities 2.01 (*SD* = 1.15), pain/discomfort 2.01 (*SD* = 1.15), and anxiety/depression 2.46 (*SD* = 1.16).

**Table 2:** Descriptive statistics of EQ-5D-5L scores and ARC scores.

Domains	Mean (SD)	Minimum	Maximum	Possible range
Mobility	1.28 (0.67)	1.00	4.00	1–5
Self-care	1.08 (0.37)	1.00	4.00	1–5
Usual activities	1.21 (0.59)	1.00	5.00	1–5
Pain/Discomfort	2.01 (1.15)	1.00	5.00	1–5
Anxiety/Depression	2.46 (1.16)	1.00	5.00	1–5
Social ARC	21.36 (3.50)	8.00	25.00	0–25
Personal ARC	21.39 (3.60)	7.00	25.00	0–25
Total ARC	42.75 (6.63)	18.00	50.00	0–50

ARC, Assessment of Recovery Capital.

Table 1 describes the five levels of self-reported 5Q-5D-5L problems by RC. Most participants with high social RC reported no problems in the mobility (84.0%), self-care (95.7%), usual activities (89.2%), and pain/discomfort (50.0%) dimensions, while 31.0% reported feeling slightly anxious/depressed, and 30.6% reported no anxiety/depression. Most participants with high personal RC reported no problems in the mobility (86.9%), self-care (98.0%), usual activities (91.8%), and pain/discomfort (53.9%) dimensions, while 32.6% reported feeling slightly anxious/depressed and 31.0% reported no anxiety/depression. Most participants with high total RC reported no mobility (85.5%), self-care (97.0%), usual activities (90.6%), and pain/discomfort (53.0%) problems. 32.5% reported feeling anxious/depressed, and 32.5% reported feeling slightly anxious/depressed.

Table 3 summarizes the most frequently reported EQ-5D-5L health states and their index values by total ARC scores. Participants self-assigned 83 EQ-5D-5L health states out of a possible 3,125 health states. The most frequent 15 states (11111, 11112, 11113, 11122, 11133, 11123, 11121, 11132, 11124, 21132, 11114, 11131, 11134, 11143, and 11135) accounted for 74.1% of participants. Only 58 participants (16.3%) self-assigned health state 11111, i.e., full health, meaning no problem on any HRQOL dimension. Our sample’s overall mean EQ-5D-5L index was 0.79 (*SD* = 0.16). Table 4 summarizes univariate and bivariate associations between participants’ sociodemographic characteristics, substance use, comorbidities, RC, and EQ-5D-5L dimensions. Overall, the highest proportion of participants reporting any problem was in the anxiety/depression dimension (78.4%), followed by pain/discomfort (55.7%), mobility (19.3%), usual activities (15.5%), and self-care (6.8%). Most participants had high social (65.4%), personal (69.0%), and total (65.6%) RC.

**Table 3:** Frequently reported EQ-5D-5L health states by total recovery capital (overall index = 0.790; *SD* = 0.16).

			Total ARC	
State	Index 0.790	Overall 355 (%)	Low 122 (%)	High 233 (%)
11111	1.000	58 (16.3)	7 (5.7)	51 (21.9)
11112	0.876	47 (13.2)	14 (11.5)	33 (14.2)
11113	0.844	36 (10.1)	11 (9.0)	25 (10.7)
11122	0.820	22 (6.2)	4 (3.3)	18 (7.3)
11133	0.800	19 (5.4)	7 (5.7)	12 (5.2)
11123	0.809	15 (4.2)	6 (4.9)	9 (3.9)
11121	0.861	11 (3.1)	1 (0.8)	10 (4.3)
11132	0.806	11 (3.1)	2 (1.6)	9 (3.9)
11124	0.669	8 (2.3)	5 (4.1)	3 (1.3)
21132	0.777	7 (2.0)	2 (1.6)	5 (2.2)
11114	0.700	6 (1.7)	4 (3.3)	2 (0.9)
11131	0.827	6 (1.7)	0 (0.0)	6 (2.6)
11134	0.661	6 (1.7)	3 (2.5)	3 (1.3)
11143	0.659	6 (1.7)	3 (2.5)	3 (1.3)
11135	0.517	5 (1.4)	4 (3.3)	1 (0.4)

ARC, Assessment of Recovery Capital.

**Table 4:** Participant’s characteristics and ARC scores by EQ-5D-5L health dimensions (*n* = 358).

Participant’s characteristics	Total n (%)	Mobility		Self-care		Usual activities		Pain/Discomfort		Anxiety/Depression	
		No problems 291(82.0)	Any problem 64 (18.0)	No problems 336 (94.7)	Any problem 19 (5.3)	No problems 307 (86.5)	Any problem 48 (13.5)	No problem 166 (46.8)	Any problem 189 (53.2)	No problem 86 (24.2)	Any problem 269 (75.8)
Age (Mean [SD])	36.0 [8.9]										
<35 years (29.3 [3.8]) 35+ years (42.6 [7.3])	172 (49.3)	**153 (53.7)**	**19 (29.7)**	165 (50.0)	7 (36.8)	**156 (51.8)**	**16 (33.3)**	**92 (57.1)**	**80 (42.5)**	46 (54.8)	126 (47.5)
	177 (50.7)	**132 (46.3)**	**45 (70.3)**	165 (50.0)	12 (63.2)	**145 (48.2)**	**32 (66.7)**	**69 (42.9)**	**108 (57.5)**	38 (45.2)	139 (52.5)
Race-ethnicity											
Hispanic	83 (23.4)	**61 (21.0)**	**22 (34.4)**	77 (22.9)	6 (31.6)	69 (22.5)	14 (29.2)	35 (21.1)	48 (25.4)	17 (19.8)	66 (24.5)
White non-Hispanic	243 (68.4)	**205 (70.4)**	**38 (59.4)**	230 (68.4)	13 (68.4)	212 (69.0)	31 (64.6)	116 (69.9)	127 (67.2)	61 (70.9)	182 (67.7)
Black/Other non-Hispanic	29 (8.2)	**25 (8.6)**	**4 (6.2)**	29 (8.6)	0 (0.0)	26 (8.5)	3 (6.2)	15 (9.0)	14 (7.4)	8 (9.3)	21 (7.8)
Sex at birth											
Male	212 (58.9)	177 (61.0)	35 (54.7)	201 (60.0)	11 (57.9)	188 (61.4)	24 (50.0)	**111 (66.9)**	**101 (53.7)**	58 (67.4)	154 (57.5)
Female	142 (40.1)	113 (39.0)	29 (45.3)	134 (40.0)	8 (42.1)	118 (38.6)	24 (50.0)	**55 (33.1)**	**87 (46.3)**	28 (32.6)	114 (42.5)
Sexual orientation											
Heterosexual	294 (82.8)	239 (82.1)	55 (85.9)	280 (83.3)	14 (73.7)	255 (83.1)	39 (81.3)	138 (83.1)	156 (82.5)	**78 (90.7)**	**216 (80.3)**
Sexual minority	61 (17.2)	52 (17.9)	9 (14.1)	56 (16.7)	5 (26.3)	52 (16.9)	9 (18.7)	28 (16.9)	33 (17.5)	**8 (9.3)**	**53 (19.7)**
Education											
High school diploma or less	176 (49.9)	144 (49.8)	32 (50.0)	167 (50.0)	9 (47.4)	157 (51.5)	19 (39.6)	84 (50.9)	92 (48.9)	42 (49.4)	134 (50.0)
Voc./some college/college	177 (50.1)	145 (50.2)	32 (50.0)	167 (50.0)	10 (52.6)	148 (48.5)	29 (60.4)	81 (49.1)	96 (51.1)	43 (50.6)	134 (50.0)
Current employment											
Unemployed	214 (60.6)	**177 (61.3)**	**37 (57.8)**	**204 (60.9)**	**10 (55.6)**	**187 (61.1)**	**27 (57.5)**	**95 (57.6)**	**119 (63.3)**	**46 (53.5)**	**168 (62.9)**
Unemployed, disabled	19 (5.4)	**6 (2.1)**	**13 (20.3)**	**14 (4.2)**	**5 (27.8)**	**8 (2.6)**	**11 (23.4)**	**4 (2.4)**	**15 (8.0)**	**2 (2.3)**	**17 (6.4)**
Employed	120 (34.0)	**106 (36.7)**	**14 (21.9)**	**117 (34.9)**	**3 (16.7)**	**111 (36.3)**	**9 (19.1)**	**66 (40.0)**	**54 (28.7)**	**38 (44.2)**	**82 (30.7)**
Marital status											
Single/Never married	220 (62.1)	**188 (64.8)**	**32 (50.0)**	**213 (63.6)**	**7 (36.8)**	**197 (64.4)**	**23 (47.9)**	112 (67.9)	108 (57.1)	55 (64.0)	165 (61.6)
Married/Common-law marriage/Committed	43 (12.2)	**33 (11.4)**	**10 (15.6)**	**38 (11.3)**	**5 (26.3)**	**35 (11.4)**	**8 (16.7)**	18 (10.9)	25 (13.2)	9 (10.5)	34 (12.7)
Separated/Divorced/Widowed	91 (25.7)	**69 (23.8)**	**22 (34.4)**	**84 (25.1)**	**7 (36.8)**	**74 (24.2)**	**17 (35.4)**	35 (21.1)	56 (29.6)	22 (25.6)	69 (25.7)
Hazardous drinking											
No or low risk/	324 (91.3)	268 (92.1)	56 (87.5)	307 (91.4)	17 (89.5)	**284 (92.5)**	**40 (83.3)**	**156 (94.0)**	**168 (88.9)**	**85 (98.8)**	**239 (88.9)**
High risk	31 (8.7)	23 (7.9)	8 (12.5)	29 (8.6)	2 (10.5)	**23 (7.5)**	**8 (16.7)**	**10 (6.0)**	**21 (11.1)**	**1 (1.2)**	**30 (11.1)**
Polydrug use											
No	87 (24.5)	76 (26.1)	11 (17.9)	83 (24.7)	4 (21.0)	**81 (26.4)**	**6 (12.5)**	**50 (30.1)**	**37 (19.6)**	**27 (31.4)**	**60 (22.3)**
Yes	268 (75.5)	215 (73.9)	53 (82.8)	253 (75.3)	15 (79.0)	**226 (73.6)**	**42 (87.5)**	**116 (69.9)**	**152 (80.4)**	**59 (68.6)**	**209 (77.7)**
Comorbid diagnosis											
No	94 (26.5)	**84 (28.9)**	**10 (15.6)**	90 (26.8)	4 (21.0)	**87 (28.3)**	**7 (14.6)**	**59 (35.5)**	**35 (18.5)**	24 (27.9)	70 (26.0)
Yes	261 (73.5)	**207 (71.1)**	**54 (84.4)**	246 (73.2)	15 (79.0)	**220 (71.7)**	**41 (85.4)**	**107 (64.5)**	**154 (81.5)**	62 (72.1)	199 (74.0)
MOUD duration (MEAN [SD])	1.2 [1.7]	**1.1 [0.1]**	**1.7 [2.7]**	1.2 [1.7]	1.5 [0.2]	**1.1 [1.4]**	**1.8 [2.8]**	1.2 [1.4]	1.3 [1.9]	1.0 [1.3]	1.3 [1.8]
Social ARC scores											
Low	123 (34.6)	96 (33.0)	27 (42.2)	114 (33.9)	9 (47.4)	**100 (32.6)**	**23 (47.9)**	**50 (30.2)**	**73 (38.6)**	**15 (17.4)**	**108 (40.1)**
High	232 (65.4)	195 (67.0)	37 (57.8)	222 (66.1)	10 (52.6)	**207 (67.4)**	**25 (52.1)**	**116 (69.9)**	**116 (61.4)**	**71 (82.6)**	**161 (59.9)**
Personal ARC scores											
Low	110 (31.0)	**78 (26.8)**	**32 (50.0)**	**96 (28.6)**	**14 (73.7)**	**82 (26.7)**	**28 (58.3)**	**34 (20.5)**	**76 (40.2)**	**10 (11.6)**	**100 (37.2)**
High	245 (69.0)	**213 (73.2)**	**32 (50.0)**	**240 (71.4)**	**5 (26.3)**	**225 (73.3)**	**20 (41.7)**	**132 (79.5)**	**113 (59.8)**	**76 (88.4)**	**169 (62.8)**
Total ARC scores											
Low	122 (34.4)	**91 (31.3)**	**31 (48.4)**	**109 (32.3)**	**13 (68.4)**	**95 (30.9)**	**27 (56.3)**	**42 (25.3)**	**80 (42.3)**	**10 (11.6)**	**112 (41.6)**
High	233 (65.6)	**200 (68.7)**	**33 (51.6)**	**227 (67.6)**	**6 (31.6)**	**212 (69.1)**	**21 (43.7)**	**124 (74.7)**	**109 (57.7)**	**76 (88.4)**	**157 (58.4)**

Voc., Vocational/Technical Diploma. Differences in counts result from missing data. Bolded values indicate statistical significance at *p* < 0.1.

### 3.1 Mobility problems

In Table 4, significant bivariate associations were age, race/ethnicity, employment, marital status, comorbid diagnoses, MOUD duration, and personal and total RC. Most participants with mobility problems had high total RC (51.6%), while half had high personal RC. Multivariable logistic regression analyses of EQ-5D-5L dimension by participant factors are summarized in Table 5. Age 35+ years (Model 1: aOR = 2.11, CI = 1.13–3.95; Model 2: aOR = 2.07, CI = 1.141–3.85), comorbid diagnoses (Model 1: aOR = 2.23, CI = 1.04–4.78, Model 2: aOR = 2.20, CI = 1.04–4.69), and low personal RC (Model 1: aOR = 0.43, CI = 0.24–0.76) were associated with mobility problems. There were no significant associations between mobility problems and social and total RC.

**Table 5:** Covariate adjusted multivariable logistic regression to predict any problems in each EQ-5D-5L health state by ARC scores and participants’ characteristics.

Any problem	Model 1: mobility and ARC domains			Model 2: mobility and total ARC		
	aOR	95% CI		aOR	95% CI	
35+ years (vs. 18–34 years)	**2.11**	**1.13**	**3.95**	**2.07**	**1.11**	**3.85**
Racial/Ethnic minority (vs. non-Hispanic White)	1.56	0.84	2.89	1.59	0.87	2.92
Employed (vs. unemployed)	0.60	0.31	1.19	0.59	0.30	1.15
Married/Committed (vs. single/never married)	1.51	0.65	3.51	1.58	0.69	3.65
Separated/Divorced/Widowed (vs. single/never married)	1.26	0.64	2.49	1.32	0.68	2.56
Comorbid diagnosis (vs. none)	**2.23**	**1.04**	**4.78**	**2.20**	**1.04**	**4.69**
MOUD duration (years)	1.12	0.96	1.31	1.13	0.97	1.32
High personal ARC (vs. low personal ARC)	**0.43**	**0.24**	**0.76**	–	–	–
High total ARC (vs. low total ARC)	–	–	–	0.59	0.33	1.05

Any problem	Model 3: self-care and ARC domains			Model 4: self-care and total ARC		
	aOR	95% CI		aOR	95% CI	
Employed (vs. unemployed)	0.41	0.11	1.53	0.40	0.11	1.45
Married/Committed (vs. single/never married)	**4.16**	**1.14**	**15.19**	**4.57**	**1.27**	**16.41**
Separated/Divorced/Widowed (vs. single/never married)	2.57	0.79	8.32	2.58	0.82	8.07
High personal ARC (vs. low personal ARC)	**0.13**	**0.04**	**0.41**	–	–	–
High total ARC (vs. low total ARC)	**–**	**–**	**–**	**0.20**	**0.07**	**0.60**

Any problem	Model 5: usual activities and ARC domains			Model 6: usual activities and total ARC		
	aOR	95% CI		aOR	95% CI	
35+ years (vs. 18–34 years)	1.83	0.88	3.80	1.63	0.81	3.30
Employed (vs. unemployed)	0.51	0.23	1.14	0.48	0.21	1.05
Married/Committed (vs. single/never married)	1.61	0.62	4.17	1.78	0.70	4.55
Separated/Divorced/Widowed (vs. single/never married)	1.26	0.58	2.74	1.36	0.64	2.89
High risk for hazardous drinking (vs. no-low risk)	1.79	0.67	4.83	1.64	0.62	4.33
Polydrug use (vs. none)	1.55	0.59	4.07	1.49	0.58	3.87
Comorbid diagnosis (vs. none)	**2.61**	**1.02**	**6.66**	**2.54**	**1.01**	**6.40**
MOUD duration (years)	1.13	0.96	1.33	1.14	0.97	1.34
High social ARC (vs. low-moderate social ARC)	1.35	0.59	3.05	–	–	–
High personal ARC (vs. low personal ARC)	**0.25**	**0.11**	**0.57**	–	–	–
High total ARC (vs. low total ARC)	–	–	–	**0.43**	**0.22**	**0.83**

Any problem	Model 7: pain/discomfort and ARC domains			Model 8: pain/discomfort and total ARC		
	aOR	95% CI		aOR	95% CI	
35+ years (vs. 18–34 years)	**1.58**	**1.00**	**2.51**	1.50	0.95	2.37
Female (vs. male)	**1.77**	**1.09**	**2.88**	**1.79**	**1.11**	**2.90**
Employed (vs. unemployed)	0.73	0.45	1.20	0.72	0.44	1.18
Polydrug use (vs. none)	1.63	0.94	2.83	1.63	0.95	2.80
High risk for hazardous drinking (vs. no-low risk)	1.72	0.72	4.09	1.58	0.67	3.74
Comorbid diagnosis (vs. none)	**2.33**	**1.39**	**3.91**	**2.26**	**1.36**	**3.77**
High social ARC (vs. low social ARC)	1.29	0.71	2.34	–	–	–
High personal ARC (vs. low personal ARC)	**0.37**	**0.20**	**0.68**	–	–	–
High total ARC (vs. low total ARC)	–	–	–	**0.55**	**0.34**	**0.90**

Any problem	Model 9: anxiety/depression and ARC domains			Model 10: anxiety/depression and total ARC		
	aOR	95% CI		aOR	95% CI	
Employed (vs. unemployed)	0.60	0.35	1.01	0.61	0.36	1.03
Sexual minority (vs. heterosexual)	**2.72**	**1.19**	**6.22**	**2.49**	**1.09**	**5.70**
Polydrug use (vs. none)	1.26	0.70	2.27	1.28	0.71	2.30
High social ARC (vs. low social ARC)	0.53	0.26	1.06	–	–	–
High personal ARC (vs. low personal ARC)	**0.33**	**0.15**	**0.73**	–	–	–
High total ARC (vs. low total ARC)	–	–	–	**0.20**	**0.10**	**0.41**

aOR, Adjusted Odds Ratio; 95% CI, 95% Confidence Interval; ARC, Assessment of Recovery Capital. Hazardous drinking was excluded from the anxiety/depression regression models due to the small cell count. Bolded values indicate statistical significance at *p* < 0.05.

### 3.2 Self-care problems

In Table 4, significant bivariate associations were employment, marital status, and personal and total RC. Most participants who reported problems conducting self-care had low personal (73.7%) and total (68.4%) RC. In Table 5, marital status- married/common-law marriage/committed relationship (Model 3: aOR = 4.16, CI = 1.14–15.19; Model 4: aOR = 4.57, CI = 1.27–16.41), low personal RC (Model 3: aOR = 0.13, CI = 0.04–0.41), and total RC (Model 4: aOR = 0.20, CI = 0.07–0.60) were associated with problems conducting self-care. There was no significant association between social RC and self-care problems.

### 3.3 Usual activities problems

In Table 4, significant bivariate associations were age, employment, marital status, hazardous drinking, polydrug use, comorbid diagnoses, MOUD duration, and social, personal, and total RC. In Table 5, most participants who reported problems conducting usual activities had high social RC (67.4%) and low personal (58.3%) and total (56.3%) RC. Comorbid diagnosis (Model 5: aOR = 2.61, CI = 1.02–6.66, Model 6: aOR = 2.54, CI = 1.01–6.40) and low personal (Model 5: aOR = 0.25, CI = 0.11–0.57) and total (Model 6: aOR = 0.43, CI = 0.22–0.83) RC were associated with problems conducting usual activities. There was no significant association between social RC and the usual activities dimension.

### 3.4 Pain/discomfort dimension

In Table 4, significant bivariate associations were age, sex at birth, employment, hazardous drinking, polydrug use, comorbid diagnoses, and social, personal, and total RC. Most participants who reported pain/discomfort problems had high social (61.4%), personal (59.8%), and total (57.7%) RC. In Table 5, age 35 + years (Model 7: aOR = 1.58, CI = 1.00–2.51), female sex (Model 7: aOR = 1.77, CI = 1.09–2.88; Model 8: aOR = 1.79, CI = 1.11–2.90), comorbid diagnoses (Model 7: aOR = 2.33, CI = 1.39–3.91, Model 8: aOR = 2.26, CI = 1.36–3.77), and low personal (Model 7: aOR = 0.37, CI = 0.20–0.68) and total (Model 8: aOR = 0.55, CI = 0.34–0.90) were associated with pain/discomfort problems. There were no significant associations between social RC and pain/discomfort problems.

### 3.5 Anxiety/depression dimension

In Table 4, significant bivariate associations were sexual orientation, employment, hazardous drinking, polydrug use, and social, personal, and total RC. Most participants who reported anxiety/depression problems had high social (59.9%), personal (62.8%), and total (58.4%) RC. In Table 5, sexual minority orientation (Model 9: aOR = 2.72, CI = 1.19–6.22; Model 10: aOR = 2.49, CI = 1.09–5.70) and low personal (Model 9: aOR = 0.33, CI = 0.15–0.73) and total (Model 10: aOR = 0.20, CI = 0.10–0.41) RC were associated with anxiety/depression problems. There were no significant associations between social RC and anxiety/depression problems. Hazardous drinking was excluded from the regression models due to the small cell size.

## 4 Discussion

To the best of our knowledge, this is the first study to examine the associations between RC and EQ-5D-5L HRQOL, described as frequencies of reported problems across five dimensions (mobility, self-care, usual activities, pain/discomfort, and anxiety/depression), among recovery residents taking MOUD in the United States. Our findings add new evidence to the growing literature on RC and HRQOL among recovery residents with OUD. We identified the health dimensions most affected by OUD and comorbid health conditions, summarized the frequency of problems across each dimension, measured the levels of RC and HRQOL problems, examined their associations, and identified strong predictors of poor HRQOL. Our findings can improve our understanding of RC and HRQOL among recovery residents with OUD. With improved understanding, health planners, recovery home administrators/operators, and policy-makers can strengthen recovery residence-based care systems through resource allocation and prioritization.

Most of our study participants self-assigned low HRQOL. Participants 35 years and older were more likely to report mobility and pain/discomfort problems than younger participants. Our findings are concerning because this is a younger sample self-reporting mobility and pain/discomfort problems, contrasting with research that demonstrated mobility problems and chronic pain are common among older adults with OUD (53, 54) and the general population (55, 56), and predictors of SUDs in older adults (57).

Our findings indicate that recovery residents with OUD who report mobility and pain/discomfort problems require special attention for several reasons. First, the most frequently reported comorbidities in our sample were mental health, respiratory, neurological, cardiovascular, and musculoskeletal conditions (Supplementary Table S1), and having either of these comorbidities is associated with mobility problems, pain/discomfort problems, and problems conducting usual activities. Second, individuals with chronic pain, respiratory, neurological, cardiovascular, and musculoskeletal/degenerative diseases are frequently prescribed opioids (55, 58–60), often misuse prescription opioids (61–63), and self-medicate with illicit drugs, resulting in SUDs, including OUD (64, 65). Furthermore, mobility problems are associated with mental illness, including anxiety and depression, increased risk of falls, and cardiovascular and respiratory conditions (66). Finally, the economic cost of pain ($560 billion to $635 billion), OUD ($471 billion), fatal opioid overdose ($550 billion), injury due to fatal ($754 million) and non-fatal ($50 billion) falls, and mental and chronic health conditions, including musculoskeletal, cardiovascular, and respiratory diseases (an estimated 90% of the United States $4.1 trillion yearly healthcare expenditure), which are attributed to direct healthcare costs, lost wages, impaired QOL, and value of statistical life, cost the United States trillions of dollars, annually (67–71).

Recovery housing provides individuals with SUDs with ongoing support, structure, and life skills building, such as healthy eating and regular exercising, to improve their physical health. Recovery housing-based systems can be leveraged to link residents with interdisciplinary healthcare professionals to provide individualized treatment for their comorbid mental and physical health conditions, including pharmaceutical and non-pharmaceutical pain management methods. The high rates of self-reported mobility and pain/discomfort problems increase residents’ risks for other comorbid conditions, polysubstance use, and relapse, underscoring the need to screen residents for pain and mobility problems and continuously monitor polysubstance use and safety to prevent opioid-related overdose deaths. Also, recovery housing staff should be trained to assess residents’ functional limitations.

We found that female residents were more likely to report pain/discomfort problems than male residents, consistent with research that demonstrated pain prevalence and severity appear higher among women (72, 73). Although the precise underlying mechanisms that drive this disparity in pain level are obscure, it has been proposed that interrelated biological and psychosocial factors may contribute to these disparities (73–79). For instance, sex differences might exist in how the opioid receptors are activated in response to pain. Further, men and women are differently socialized to talk about and cope with painful experiences, including adverse childhood experiences. Thus, when compared to men, women will more often employ a diverse set of coping strategies, including relying on social support, while men typically use behavioral distraction or problem-focused approaches (77, 80). There are potential advantages in providing comprehensive, integrated care models that do not place the blame on female residents experiencing pain. Rather, interventions might be needed to support recovery residences’ staff to link female residents to trauma-informed outpatient treatment programs and other services, including social support and mutual aid groups, which are central to improving substance use outcomes (81, 82). While sex differences existed in our sample only on the pain/discomfort dimension, other differences may exist. Future research with larger samples should explore sex differences across all HRQOL dimensions.

Participants who are married or in a committed relationship were more likely to report problems conducting self-care than single or never-married participants, contrary to previous research that reported married individuals have better HRQOL and health than single, divorced/separated, or bereaved individuals (83–85). This contrasting result may be because residents’ partners also engage in opioid or other substance use, highlighting the need to provide behavioral couples therapy to residents and their married or cohabiting partners.

The highest proportion of participants reporting any HRQOL problem was in the anxiety/depression dimension. Anxiety and depression frequently co-occur with OUD and are risk factors for opioid misuse and OUD (15, 17). We also found that sexual minorities were more likely to report anxiety/depression problems than heterosexuals, consistent with previous researchers’ findings that indicate sexual minorities have poorer HRQOL, and gay/bisexual men are more likely to report anxiety/depression problems than heterosexuals (86). Given that sexual minorities report worse health outcomes, including anxiety, depression, poor HRQOL, and health-related behaviors, than heterosexuals due to stigma and discrimination (86–88), culturally competent co-occurring mental health and SUDs treatment and support must be integrated into recovery residence-based systems of care.

Most participants had high total, social, and personal RC, consistent with research that reported individuals with higher RC are more likely to identify and access substance use treatment than those with lower RC (89). Low personal RC was associated with problems in all HRQOL dimensions. Similarly, low total RC was associated with HRQOL problems, except mobility problems. Personal RC includes mental and physical health and education/vocational skills (41, 42). Indeed, most participants had attended some college or had a vocational/technical diploma or college degree and reported comorbid mental and physical diagnoses, which may account for the differences in HRQOL by personal RC observed. The presence of comorbid mental and physical conditions may be due to OUD or exacerbated by OUD, which decreases HRQOL (12). Future research should identify and assess the effects of all types of personal RC on HRQOL so recovery staff will better understand how to strengthen residents’ RC to support their recovery and improve their HRQOL.

The logistic regression model suggests that social RC is not associated with any HRQOL dimension. Our finding is novel compared to previous researchers that reported significant associations between social RC and HRQOL (43, 45, 90). White and Cloud (42) suggest that long-term recovery is predicted by social and personal RC, with the impact of low personal RC being regulated by high social RC. Contrarily, using HRQOL as a proxy for long-term recovery, personal RC is a stronger predictor of long-term recovery, and high personal RC appears to regulate the impact of low social RC in our sample. Nonetheless, recovery homes can provide a safe and supportive environment for residents with low social RC to connect with their peers to improve their health, well-being, and recovery from OUD. This is critical as social capital, such as social support and connectedness obtained from social networks and supportive relationships, are protective against substance use, anxiety, and depression (41, 42, 91–93).

Our results should be interpreted with caution due to several limitations. First, our analysis has a cross-sectional design, limiting our ability to assess causal relationships. Second, most participants were non-Hispanic White and resided in levels II and III certified recovery homes across five cities in Texas. Therefore, our findings may not be generalizable to other individuals with OUD. Furthermore, our analysis of RC and HRQOL relied on self-reported data, which may introduce social desirability and recall bias. Also, with a larger sample size, potentially meaningful differences that were not statistically significant in our sample might have been identified, e.g., sex differences across all HRQOL dimensions and not just the pain/discomfort dimension alone. Finally, the ARC scale assesses only two of the five RC domains; thus, total RC in our study refers to only the social and personal resources that residents can draw upon to initiate and sustain their recovery from OUD. Nonetheless, personal RC is the stronger predictor of HRQOL and long-term recovery among recovery residents with OUD.

## 5 Conclusion

Our analysis of data from individuals taking MOUD and residing in levels II and III recovery homes indicates that most residents have high RC. However, personal RC is a stronger predictor of HRQOL than social RC. Our findings underscore the importance of examining each RC domain and increasing RC to influence the HRQOL of individuals with OUD. We also found that comorbid conditions, including mental illness, respiratory, neurological, musculoskeletal, and cardiovascular diseases, are highly prevalent and negatively impact HRQOL. Our results emphasize the need for policymakers to support the integration of MOUD and treatment for comorbid conditions, especially mental illness and chronic physical health conditions that result in pain, mobility issues, and problems conducting usual activities. Recovery is multidimensional and lifelong; thus, researchers and recovery residence administrators/staff should be trained to identify and understand RC and its influences on HRQOL to strengthen residents’ existing or develop new RC. Recovery providers can support residents’ recovery to improve opioid use outcomes and HRQOL by participating in intervention design, interprofessional models of care, and policies. This study adds to the HRQOL and RC literature by identifying the most frequently reported problems in five EQ-5D-5L HRQOL dimensions and predictors of poor HRQOL, i.e., female sex, sexual minority identity, married status, comorbid conditions, and low recovery capital, among recovery residents with OUD.

## Data availability statement

The original contributions presented in the study are included in the article/Supplementary material, further inquiries can be directed to the corresponding author.

## Ethics statement

The studies involving humans were approved by the Institutional Review Board of the University of Texas Health Science Center at Houston, Committee for the Protection of Human Subjects (CPHS). The studies were conducted in accordance with the local legislation and institutional requirements. The participants provided their written informed consent to participate in this study.

## Author contributions

EO: Conceptualization, Data curation, Formal analysis, Methodology, Software, Validation, Visualization, Writing – original draft, Writing – review & editing. SM: Conceptualization, Funding acquisition, Investigation, Supervision, Writing – review & editing. VS: Conceptualization, Supervision, Writing – review & editing. CM: Conceptualization, Supervision, Writing – review & editing. KG: Writing – review & editing, Investigation, Project administration. JW: Conceptualization, Funding acquisition, Investigation, Methodology, Resources, Software, Supervision, Writing – review & editing.

## Supplementary material

### Abbreviations

aOR, Adjusted Odds Ratio; 95% CI, 95% Confidence Interval; HIPAA, Health Insurance Portability and Accountability Act; HRQOL, Health-Related Quality of Life; MOUD, Medication for Opioid Use Disorder; OUD, Opioid Use Disorder; RC, Recovery Capital; SUDs, Substance Use Disorders.
