# Supplementary material for: Health-related quality of life and recovery capital among recovery residents taking medication for opioid use disorder in Texas
Source: Front Public Health. 2023 Nov 20;11:1284192. doi: 10.3389/fpubh.2023.1284192 (PMC10694473; doi:10.3389/fpubh.2023.1284192)
Supplement: Supplementary file 1 [file Table_1.DOCX]

Supplemental Table 1: Frequently reported substance use and comorbid conditions

|  | Total n (%) |
| --- | --- |
| **Polydrug use** |  |
| Street opioids | 197 (55.0) |
| Amphetamines | 162 (45.3) |
| Methamphetamine | 151 (42.2) |
| Benzodiazepines | 139 (38.8) |
| Marijuana | 134 (37.5) |
| Prescription opioids | 93 (26.0) |
| Cocaine | 87 (24.3) |
| **Mental health conditions** |  |
| Depression | 53 (14.8) |
| Anxiety | 51 (14.3) |
| Bipolar | 30 (8.4) |
| Other | 40 (11.2) |
| **Respiratory conditions** |  |
| Asthma | 41 (11.5) |
| Bronchitis | 39 (10.9) |
| Pneumonia | 34 (9.5) |
| Other (e.g., emphysema, tuberculosis) | 26 (7.3) |
| **Neurological conditions** |  |
| Migraines | 44 (12.3) |
| Memory loss | 33 (9.2) |
| Other (e.g., epilepsy, traumatic brain injury) | 7 (2.0) |
| **Cardiovascular conditions** |  |
| High blood pressure | 56 (15.6) |
| High cholesterol | 11 (3.1) |
| Stroke and heart disease | 6 (1.7) |
| **Musculoskeletal conditions** |  |
| Bone fractures | 33 (9.2) |
| Arthritis | 28 (7.8) |
| Osteoporosis | 6 (1.7) |
| Other (e.g., chronic pain, osteomyelitis) | 5 (1.4) |

Note: Differences in counts result from missing data.
